# Stimuli‐Responsive Phosphorus‐Based Polymers

**DOI:** 10.1002/ejic.201801077

**Published:** 2018-12-10

**Authors:** Ian Teasdale

**Affiliations:** ^1^ Institute of Polymer Chemistry Johannes Kepler University Linz Altenberger Straße 69 4040 Linz Austria

**Keywords:** Polyphosphoesters, Phosphazenes, Phosphorus, Polymers, Synthesis design

## Abstract

This microreview details recent developments in stimuli‐responsive polymers with phosphorus in the main‐chain, in particular polyphosphazenes and polyphosphoesters. The presence of phosphorus in the polymers endows unique properties onto the macromolecules, which can be utilized for the preparation of materials capable of physically responding to specific stimuli. Achieving the desired responsiveness has been much facilitated by recent developments in synthetic polymer chemistry, in particular controlled synthesis and backbone functionalization phosphorus‐based polymers, in order to achieve the required properties and hence responsiveness of the materials. The development of phosphorus‐based polymers which respond to the most important stimuli are discussed, namely, pH, oxidation, reduction, temperature and biological triggers. The polymers are placed in the context not just of each other but also with reference to state‐of‐the‐art organic polymers.

## 1. Introduction

While the importance of polymers both as commodity and as high‐performance materials is long established, in recent decades a better fundamental understanding of structure property relationships has been combined with vast improvements in controlled polymerization methods. This has allowed for the preparation of materials with evermore finely tuned macromolecular structures and with it, more advanced and precise tailor‐made properties. Among these developments, stimuli‐responsive polymers,^1^ also sometimes referred to as “smart polymers”, represent a group of macromolecular materials which can undergo reversible or irreversible chemical or physical changes in response to external stimuli. Stimuli commonly studied include light^2^ pH,^3^ temperature,^4^ oxidation,^5^ and magnetic fields.^6^ Applications of stimuli‐responsive polymers include use as chemo‐ and biosensors,^7^ as chemomechanical actuators,^8^ as well as widespread roles in biomedicine, for example the selective release of drugs in specific biological environments.

Phosphorus‐based polymers, while much less prevalent than their carbon‐based analogues, are of widespread interest in a number of areas in polymer science. Indeed since the biopolymers DNA and RNA are macromolecules with phosphorus in the main‐chain and poly(phosphate) and its cleavage products are responsible for a variety of essential pathways,^9^ it could be stated that life would be impossible without phosphorus‐based polymers. The most common families of phosphorus‐based synthetic polymers are polyphosphoesters and polyphosphazenes (Figureà1). Interestingly, both families of polymers show a propensity to undergo degradation to phosphates^10^ via hydrolysis at the main‐chain phosphorus atom. The substituents on the phosphorus determine the rate of hydrolysis hence polymers with a wide range of different degradation rates are accessible. This tunable degradation to biocompatible degradation products (phosphate salts are prevalent in biological media) has driven much interest in their application in medical applications.^11^ From a synthesis perspective, the varied substitution and multivalency possible at a phosphorus center in a polymer main‐chain leads to a high density of functional groups and the ease of functionalization allows the facile tuning of the chemical and mechanical properties of the polymers.

**Figure 1:**
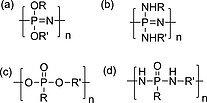
Figure 1. Generic chemical structures of some phosphorus main‐chain polymers (a) polyalkoxyphosphazenes, (b) polyaminophosphazenes, (c) polyphosphoesters, and (d) polyphosphoramidates.

Phosphorus‐containing moieties can be readily incorporated onto organic polymers, for example via vinyl monomers with phosphonic acid or acidic phosphates, or alternatively phosphorus can be incorporated as branching units in well‐defined dendrimers, with both routes leading to rich families of functional polymers. The reader's attention is directed elsewhere for a review of these substances (see[11b] and^12^respectively) with the focus in this microreview on linear high polymers with phosphorus in the main chain. Although synthetic polymers with phosphorus in the main‐chain have been known for many years, only recently have methods have been developed which allow controlled polymerization and advanced structural control. Indeed, today, controlled polymerization procedures are readily available for the most common phosphorus main‐chain polymers, polyphosphazenes,^13^ polyphosphoesters,^14^ and poly(phosphorodiamidate)s,^15^ while the spectrum of methods available is narrow, they can be regarded as equivalent to modern organic polymerization methods in terms of practical handling and the quality of the control available.

## 2. pH‐Responsive Polymers

One of the most simple but effective external stimuli for responsive polymers is pH, which can be easily varied and is particularly useful for biomedical applications due to the prevalence of pH gradients in biological systems. The following three sections hence describe some recently developed systems in which pH is used firstly to cleave macromolecules with phosphorus in the main‐chain, thus degrading the polymers and secondly to cleave active small molecules from macromolecules. Thirdly a few examples are described whereby non‐degrading structural changes are induced upon pH adjustment.

### 2.1. pH‐Stimulated Degradation

In applications such as the triggered release of pharmaceuticals, polymers which are stable at neutral pH but undergo rapid backbone cleavage at lower pH values (pH 4–6) are of particular significance, for example as polymer therapeutics or for the encapsulation of active pharmaceutical ingredients. There are numerous examples of cleavable organic polymers incorporating acid‐labile moieties, for example acid‐sensitive acetal moieties^16^ or orthoesters^17^ in the main‐chain. However, the incorporation of acid‐cleavable moieties is most commonly achieved via step‐growth mechanisms, hence molecular weights are difficult to control and dispersities broad. Moreover, the concurrent chemical functionality of the polymer is limited by choice of monomers. These are two difficulties which researchers have attempted to overcome by exploiting phosphorus‐based systems, which allow a wider range of functional moieties and controlled synthesis routes.

The polyphosphazene backbone shows an inherent tendency to hydrolyze, a process which has been confirmed in numerous studies to be significantly accelerated under acidic conditions.^18^ The post‐polymerization functionalization of the backbone thus allows facile access to a wide range of pH sensitive polymers. For example, Andrianov et al. showed that water soluble *N*‐ethylpyrrolidone substituted polyphosphazenes undergo accelerated degradation at pH 3 compared to pH 7.4.^19^ Meanwhile our group has prepared a series of water‐soluble bottle‐brush type polyphosphazenes with pendant Jeffamine (amine‐capped ethylene oxide/ propylene oxide) oligomers attached via an amino acid spacer. These polymers were shown to have pH stimulated degradation profiles that was significantly faster at pH 5 than pH 7, with further acceleration when the pH is reduced to 2. A similar pattern was observed for polyphosphazene‐based dendritic polyols^20^ (Figureà2b), as well as solid porous matrices.^21^

**Figure 2:**
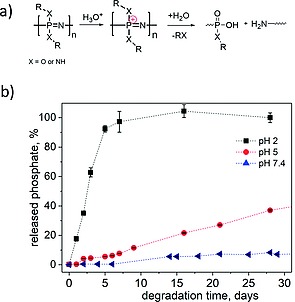
Figure 2. (a) The proposed mechanism for pH‐catalyzed polyphosphazene chain cleavage and (b) Rate of phosphate release from the main‐chain of polyphosphazene‐based polyols as observed by a photometric phosphate assay. Adapted from ref.^20^

The reason for the selective backbone cleavage of polyphosphazenes at acidic pH values is due to protonation of the nucleophilic nitrogen in the backbone, which facilitates nucleophilic attack of water and hence hydrolysis of the P–N bonds. The proposed degradation mechanism is shown in Figure 2a. The final degradation products of the backbone consist of ammonium and phosphate ions. Interestingly Andrianov et al. have shown polyphosphazenes have a higher stability at basic pH, both for polyaminophosphazenes,[18a] as well as for poly[di(carboxylatophenoxy)phosphazene]s,^22^ a strong contrast to, for example, polyesters, the most widely reported family of degradable synthetic polymers. The nature of the polyphosphazene substituents must however also be taken into account, for example polyphosphazenes with ester groups in their side chains, which may also cleave at basic pH values due to cleavage of the ester exposing carboxylic acid functionalized polyphosphazene main‐chains which are in turn susceptible to rapid main‐chain hydrolysis.^23^

**Figure 3:**
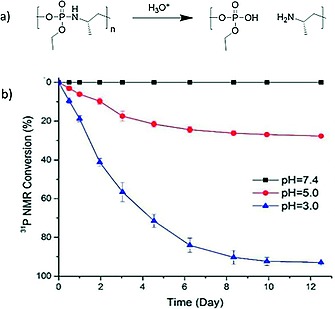
Figure 3. (a) The acid‐triggered degradation mechanism of polyphosphoramidates and (b) the release of phosphate upon backbone cleavage as monitored by ^31^P NMR spectroscopy at different pH values. Adapted with permission from ref.^26^ Copyright 2017 American Chemical Society.

Polyphosphoesters are also well known to undergo hydrolytic main‐chain cleavage. In contrast to polyphosphazenes, a significantly faster rate of cleavage is usually observed under basic conditions,^24^ whereas under acidic conditions cleavage of pendant methyl moieties tends to occur first, followed by slower main‐chain degradation.^24^ Interestingly, poly(phosphorodiamidate)s, recently prepared via olefin metathesis polymerization,^25^ are reported to be quite stable under basic conditions but can be degraded by acidic hydrolysis as a result of their having P–N bonds as oppose to P‐O bond in the main‐chain. Furthermore, recently polyphosphoramidates have also been designed to undergo rapid main‐chain cleavage at acidic pH values. The incorporation of acid labile phosphoramidate bonds was achieved via controlled organobase‐catalyzed ring‐opening polymerization (ROP) of a newly prepared oxazaphospholidine monomer (Figureà3a).^26^ The presence of P–N bonds in the polymer backbone resulted in a rapid polymer degradation when exposed to acidic pH values (Figure 3b).

### 2.2. pH‐Triggered Release of Small Molecules

Acid‐cleavable polyphosphoramidates have also been investigated in nanoformulations, for example with the anticancer drug campthothecin.^27^ A pH selective release of the drug payload was observed at pH 5 due to the acid‐triggered cleavage of the polymers and hence disintegration of the nanoparticles. Similarly, polyphosphazenes have been investigated for non‐covalent nanoformulations and drug encapsulation.[11a] For example, inulin loaded degradable matrices based on 50 % ethyl glycinato and 50 % *p*‐methylphenoxy substituents which show a pH dependent release profile.^28^ Although practical and relatively simple, drug encapsulation in such systems rely on bioerosion of the polymer matrix for payload release and hence release rates can be difficult to control. Clearly a tricky balance is required between matrix stability and the requirement for rapid drug release. An alternative approach is to decouple the release process from the degradation process to design polymeric drug carriers which undergo a rapid, specific triggered release followed by a slower, complete degradation to small molecules to be cleared from the body and hence be safe for clinical application.^29^

For the design of triggered‐release polymeric systems, many drug linker strategies can be adapted from well‐established organic or indeed organometallic prodrug systems.^30^ Since controlled structures and timely degradation rates is essential for any polymers used for intravenous injection,^31^ polyphosphazenes^32^ and polyphosphoesters^33^ are suitable candidates for such a strategy. This can be achieved either through non‐covalent loading in nanostructures with cleavable cross‐links or alternatively via macromolecular prodrugs, whereby the prodrug is covalently bound to the polymer. For example using acid‐cleavable acetal moieties as cross‐linkers for polyphosphoesters has been used to prepare drug‐loaded core‐cross‐linked micelles^34^ to prepare promising materials for targeted cancer chemotherapy.

Alternatively, hyperbranched polyphosphoesters, with their excellent aqueous solubility and ample end‐groups for functionalization have also been highly successfully applied with a wide range of release systems in this field.^36^ As demonstrated with the model drug chlorambucil, attachment of the active drug via ester moieties on the hydroxyl end‐groups^37^ provides a simple route to prepare covalently bound polymer therapeutics which can release their payload upon endocytosis. Similarly, poly(oxyethylene H‐phosphonate)s can be readily functionalized along the backbone to prepare macromolecular prodrugs.^38^ Excellent water solubility, ease of functionalization and degradability make these promising carriers, potentially as alternatives to the ubiquitous but non‐degradable polyethylene glycol‐based systems.

In a similar approach, our group, in collaboration with others recently investigated the covalent linkage of ruthenium half‐sandwich compounds with branched polyphosphazenes to prepare macromolecular prodrugs(Figureà4).^35^ The ruthenium half‐sandwich complexes show excellent activity as anticancer drugs but typically suffer from very low aqueous solubility, hence conjugation to the water‐soluble polyphosphazene carrier significantly enhances its applicability. Attachment to the polymer was achieved via amine‐ruthenium bonds, which are hydrolysable at lower pH values, thus allowing a controlled, pH‐triggered release of the active metallodrug.

**Figure 4:**
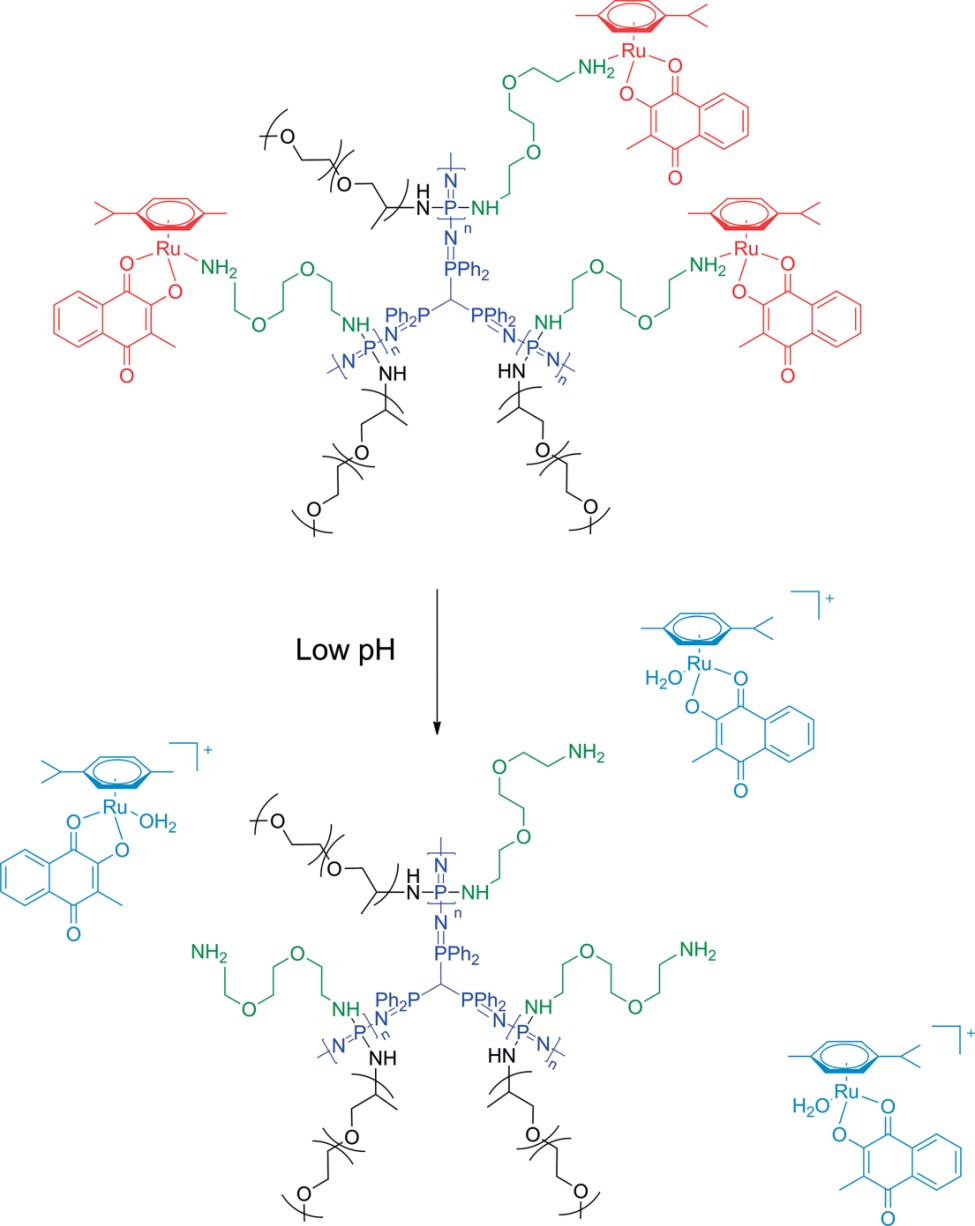
Figure 4. Branched polyphosphazenes loaded with ruthenium‐based anticancer drugs. Reproduced from ref.^35^

**Figure 5:**
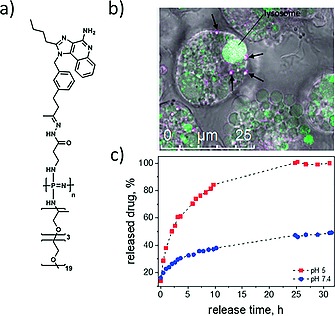
Figure 5. (a) Chemical structure of a pH sensitive imidazoquinoline macromolecular prodrug, (b) accumulation of the prodrugs in endosomes (arrows point to the endosomes), and (c) the pH selective release of the drug. Adapted from ref.^42^

Macromolecules are known to undergo cell uptake via endocytosis pathways^39^ and this is nowadays a widespread targeting tactic in polymer therapeutics.^40^ With this knowledge in mind, we recently designed polyphosphazene macromolecular prodrugs for cancer immunotherapy. A common strategy in immunotherapy is the supply of agonistic ligands to dendritic cells, which serve to stimulate the immune system.^41^ Since the TLR7/8 the receptor sites are located in the endosomes, macromolecular prodrugs were designed to transport the agonist imidazoquinoline to the required site^42^ (Figureà5a). Accumulation in endosomes could be observed via confocal microscopy of the fluorescent macromolecules (Figure 5b). Since the endosomal pH is also known to be significantly lower, a pH stimulated release was utilized (Figure 5c), in this example a hydrazone linkage between polyphosphazene and immune response modifier facilitates an endosomal, site‐specific presentation of the active ligand.

The endosomal trafficking strategy can also be used to enhance cell uptake of small molecule drugs. Our group, in collaboration with others demonstrated this with a series of Pt^IV^ prodrugs designed to undergo intracellular reduction.^43^ Through covalent attachment as pendant groups along the backbone of a water‐soluble polyphosphazene, it was possible to prepare Pt^IV^ loaded macromolecular carriers. Attachment via axial ligands meant the Pt complexes underwent simultaneous release from the macromolecule upon reduction. Due to the endocytic pathway, not accesible to the small‐molecule Pt species, a considerable 30‐fold increase in the uptake was observed for the polymer‐bound drugs. While the Pt^IV^ drug showed minimal cytotoxic effects due to its low uptake, the loaded macromolecular prodrug was highly active against a number of cancer cell lines (Figureà6). This observation was attributed to the high uptake in combination with the reduction of the Pt^IV^ prodrug to the active Pt^II^ species once exposed to the acidic endosomal environment. In vivo studies, although also promising, showed a less pronounced effect, but here the picture is more complex with other factors, not least biodistribution must also be taken into account, hence work in this area is ongoing.

**Figure 6:**
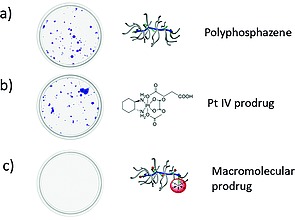
Figure 6. Visual demonstration of cell survival rates upon treatment with (a) polyphosphazene, (b) a Pt^IV^ prodrug, and (c) the same Pt^IV^ prodrug covalently bound to the polyphosphazene. While complete cell death was observed for the macromolecular prodrug, neither the polymer alone nor the prodrug alone showed significant activity against HCT colon carcinoma cells, incubated for 7 days with 5 µM of the drug. Images courtesy of Kustrim Kryeziu from data published in ref.^43^

### 2.3. pH‐Induced Morphological Changes

Selective backbone protonation of hydrolytically stable polyalkylphosphazene block copolymers in acidic media has also been used to drive morphological changes of self‐assembled systems (Figureà7).^44^ Since polyalkylphosphazenes are not sensitive to hydrolysis due to their stable P–C bonds, morphological changes occur without degradation of the polymer. A recent review article describes the advances in the field of self‐assembly of this family of polymers.^45^

**Figure 7:**
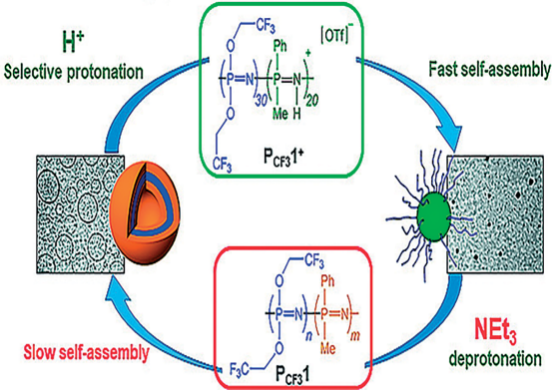
Figure 7. Selective protonation of polyphosphazene block copolymers induces rapid self‐assembly. Reproduced from ref^45^ and adapted from original work in ref.^44^.

Similarly, vesicles prepared from amphiphilic polyphosphazene with pendant tertiary amines, have been investigated for drug release systems.^46^ The structure of vesicles was disturbed through amine protonation at pH 5.5 leading to a sharp pH‐triggered drug release profile. More recently, Andrianov and co‐workers have shown that found that aqueous solutions of polyphosphazenes co‐substituted with carboxylic acid and pyrrolidone moieties undergo spontaneous self‐assembly into nanoparticulate structures in an acidic environment.^47^ The pH of the response could be tuned to match that of the endosomal environment, hence cell‐surface interactions and the intracellular delivery of protein macromolecular drugs into cancer cells were significantly enhanced.

## 3. Reduction‐Sensitive Polymers

A commonly used strategy to prepare reduction sensitive polymers is the incorporation of disulfide linkages into macromolecules, either into the main‐chain or as pendant groups^48^. For example, the covalent attachment of the anticancer drug campthothecin to a polyphosphoester via a disulfide linker (Figureà8) has been utilized to prepare a polyphosphoester‐based macromolecular prodrug that can efficiently release the drug and effectively inhibit cell proliferation.^49^ The incorporation of disulfides into the main‐chain has been successfully achieved for hyperbranched polyphosphoesters,^50^ as well as the more reduction‐sensitive selenium derivatives.^51^ This family of polymers have considerable potential in targeted drug delivery as the introduction of reductive bonds leads to polymers which are stable in the blood stream and extra‐cellular fluids but rapidly cleave in intracellular compartments such as the cytoplasm and the cell nucleus. This has for example been used for the controlled release of the anticancer drug doxorubicin.^50^

**Figure 8:**
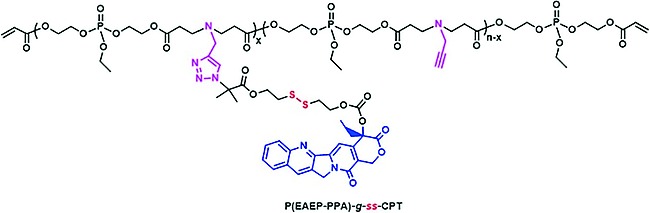
Figure 8. A polyphosphoesters with covalently bound chemotherapeutic camptothecin via a disulfide bridge for selective release in intracellular compartments. Adapted with permission from ref.^49^ Copyright 2017 American Chemical Society.

## 4. Oxidation‐Responsive Polymers

Oxidative processes also represent widely investigated stimuli which have been for responsive polymers.^52^ Polymers sensitive to oxidative stress are of special interest for a host of clinical applications. An excess of oxidative species is a common situation for a number of disorders including inflammations, has been associated with cancerous tissue and is also a marker for cardiovascular disease.^5^ In this context, polymers that cleave or degrade in response to biological concentrations of H_2_O_2_ are being investigated for the triggered release of active payloads and numerous examples have been developed, mostly based on oxidation‐sensitive arylboronic esters including, for example, dextran‐based microparticles drug carriers^53^. Further examples include self‐immolative polycarbonates^54^, as well as polyesters with pendant phenylboronic ester moieties along the backbone which initiate a “chain‐shattering” of the polymer upon contact with H_2_O_2_.^55^

While elegant chemistry, the preparation by of self‐immolating organic polymers is limited by the requirement to incorporate functional groups into the polymer main‐chain. Hence our group prepared polydichlorophosphazene and substituted the main‐chain with phenylboronic ester moieties[5b] (Figureà9). This post‐polymerization functionalization allows not only prior determination of the macromolecular chain length but also facile adjustment of the number of oxidation sensitive moieties (hence potentially cleavage rates). Furthermore the polymers can be readily co‐substituted allowing tailoring of properties of the polymers, for example aqueous solubility. The combination of controlled synthesis, tailorability and known biocompatibility of the polyphosphazene scaffold render such materials quite promising tools for oxidation‐responsive drug delivery.

**Figure 9:**
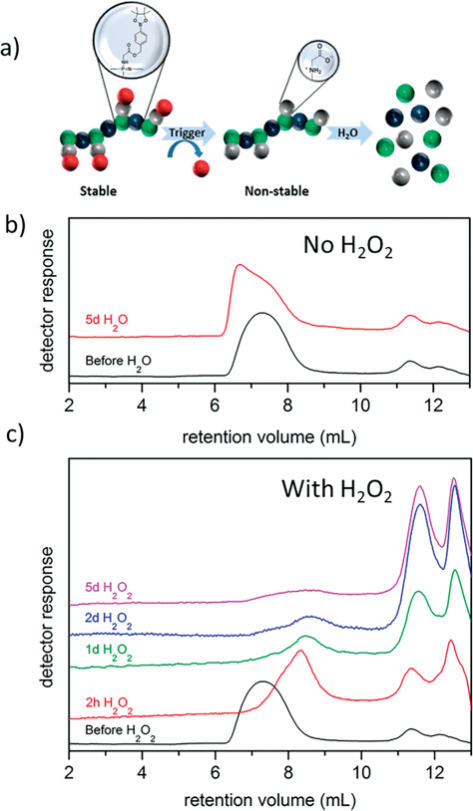
Figure 9. (a) Conceptualization of phenylboronic ester functionalized polyphosphazenes. Oxidation, cleavage, and self‐immolation of the phenylboronic ester exposes the hydrolytically sensitive polyphosphazene main‐chain triggering degradation of the polymer. (b) Exemplary SEC chromatographs in the absence of H_2_O_2_ show a stable polymer whereas (c) addition of 10 mM H_2_O_2_ causes rapid chain cleavage. Adapted from ref.[5b]

Oxidation as a stimulus is not limited to polymer cleavage processes. One interesting approach in terms of stimuli‐responsive polymers is the reaction of a organophosphorus(III) moieties in a polymer backbone with hydrogen peroxide. D. P. Gates and co‐workers have shown, for example, that when *C*‐aryl chromophores are attached to phosphorus moieties in a polymer main‐chain, the polymers underwent a switch from non‐emissive to highly emissive in response to the presence of H_2_O_2_.^56^ Such materials could clearly be highly interesting for applications in sensor technology.

## 5. Photoresponsive Polymers

The use of light as a stimulus to evoke a response in polymer materials is particularly attractive, enabling spatiotemporal control over the desired response. Such materials are highly sought after in fields such as photolithography, controlled drug release and for the preparation of advanced sensory materials.^57^ Of particular importance for these fields are polymers which respond in the visible/red region, in order to circumvent the issues of low penetration and damaging effects of UV irradiation. This can be either to remove the polymers, cleave small‐molecules from them or to show activity themselves in response to stimulation with certain wavelengths of irradiation.

### 5.1. Photocleavable Polymers

Polymers which can be cleaved or undergo backbone disassembly upon response to light in a spatiotemporal manner are of interest as positive photoresists or in controlled release applications^59^. In this context, our group has recently reported polyphosphazenes which undergo rapid degradation to small molecules upon photochemical activation with light in the visible region.^58^ This works on the basis of an hydrolytically instable poly(glycine)phosphazene backbone, in which the acid groups are caged (protected) by photochemically labile coumarin moieties (Figureà10). Irradiation exposes carboxylic acid moieties along the polymer leading to a rapid hydrolytic degradation of the polyphosphazene main‐chain. The known biocompatibility of similar polyphosphazenes,^60^ combined with the sensitivity towards light in the visible region mean these materials are particularly significant as photocleavable materials in biological applications.

**Figure 10:**
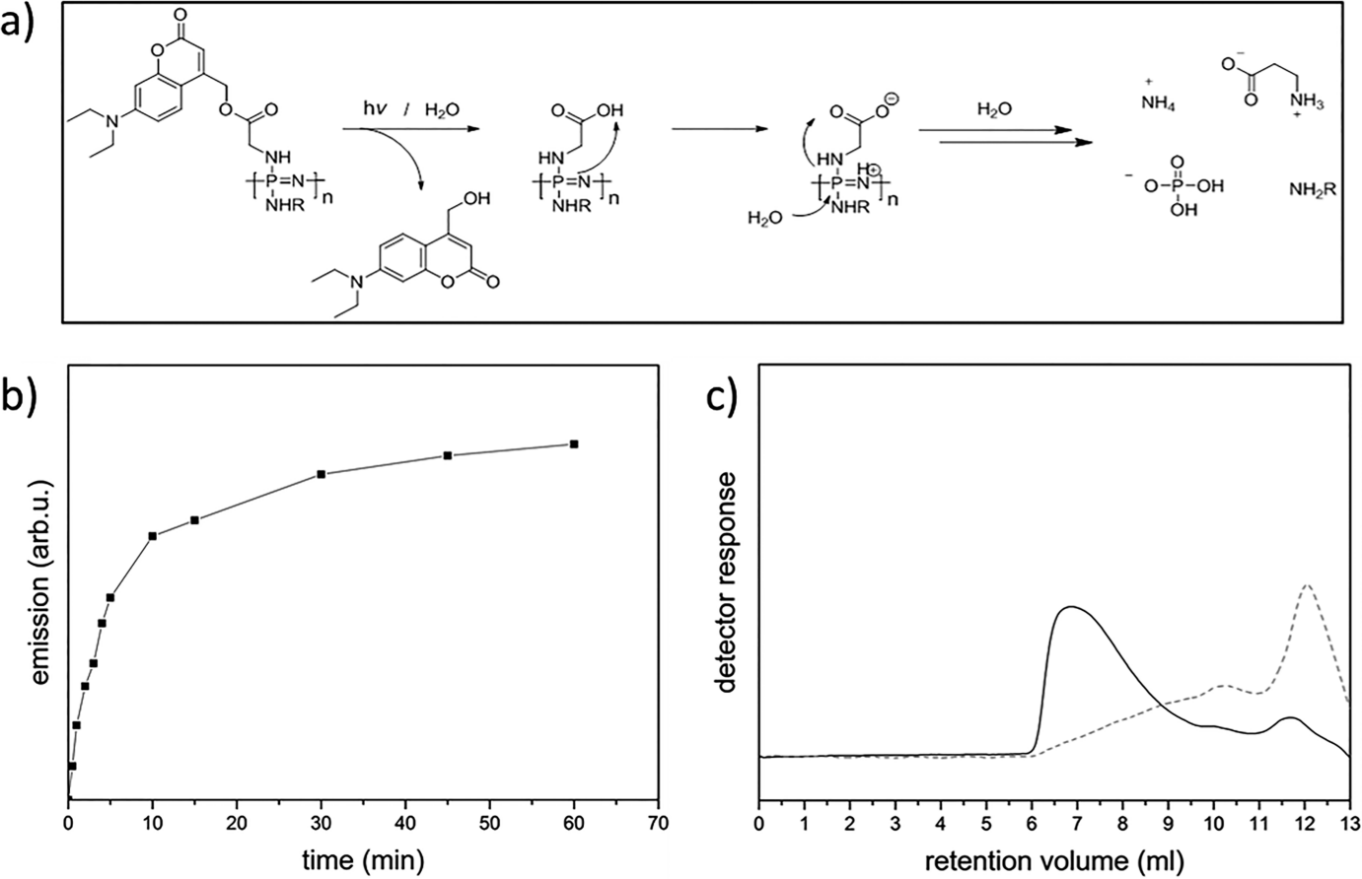
Figure 10. a) Proposed mechanism for the degradation of photocleavable polyphosphazenes. b) Progress of emission intensity of coumarin‐functionalized polyphosphazene shows the release from the polymer upon irradiation with visible light and c) SEC analysis of the polymer before and after irradiation. Adapted from ref.^58^

### 5.2. Photoactive Polymers

The optical transparency of the polyphosphazene main‐chain lies between the near‐infrared to below 220 nm^61^ and in comparison to many organic polymers, shows no propensity towards photochemical damage, assuming the organic substituents are carefully chosen. This relative stability to light and high transparency underlies their use as optical materials^62^ or indeed as photopolymers.^21^, ^63^ Meanwhile, the ease of functionalization of the polyphosphazene backbone allows it to be readily loaded with chromophores, for example Allcock and co‐workers reported the preparation film‐forming polyphosphazenes with a mixture of red, green, and blue dye molecules.^61^ The dye loading and the properties of the polymers could be varied to provide patterned tricolor filters for the development of stable, printable color filters for liquid crystal displays.

In an example from our laboratory, hypericin, a clinical photosensitizer could be conjugated to water‐soluble polyphosphazenes.^64^ Hypericin administration is severely limited due to its extremely poor aqueous solubility, but upon macromolecular conjugation, both covalently^64^ to the phosphorus via its hydroxyl moieties, as well as non‐covalently onto pyrrolidone functionalized polyphosphazenes, the water solubility could be significantly enhanced. Detailed in vitro testing of the photosensitizer conjugates showed them to be efficient inducers of cell apoptosis upon irradiation at 610 nm (Figureà11).^65^

**Figure 11:**
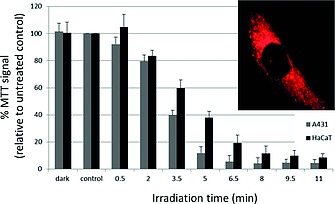
Figure 11. Light‐dependent cytotoxicity of polyphosphazene bound photosensitizers (5 µM) in A431 and HaCaT cells. Cell survival in % was related to the untreated control C_0_. Irradiation parameters: *λ*_max_ = 610 ± 10 nm; power density = 1.8 mW cm^–2^and (inset) localization of 5 µM hypericin bound polyphosphazene in primary human dermal fibroblasts after 3 hours of incubation. 1000× magnification; oil‐immersion (filter: *λ*_Ex_ = 480–500 nm and *λ*_Em_ > 570 nm). Adapted from ref.^65^ with permission from the European Society for Photobiology, the European Photochemistry Association, and The Royal Society of Chemistry.

The degradability and ease of loading means polyphosphoesters also lend themselves as macromolecular carriers for photosensitizers. Recently Yang and co‐workers have shown that the photosensitizer chlorin e6 could be loaded onto hyperbranched polyphosphoesters.^66^ Since the polyphosphoesters also incorporated acetal linkages, the materials showed double stimulus selectivity with light and pH. The pH triggered release of the photosensitizer upon endocytosis was shown to enhance the intracellular ROS generation and hence the therapeutic efficacy of the carriers.

## 6. Polymers Responsive to Enzymatic Cleavage

It may be anticipated that polymers containing phosphate in the main‐chain would be susceptible to enzymatic cleavage, and it is often cited as a characteristic of polyphosphoesters.[10b] However, genuine studies of selective enzymatic degradation are relatively rare, with most focusing on the more prominent hydrolytic degradation pathways. For example, while it is reported that polyphosphoester‐polycaprolactone (PCL) block copolymers undergo enzymatic degradation in the presence of the ester cleaving pseudomonas lipase enzyme, subsequent studies have shown that only the PCL component of these structures is selectively degraded^67^, with the polyphosphoesters degrading predominantly by hydrolysis. It has, however, been shown that alkaline phosphatase, an important enzyme produced in bone and liver cells, catalyzes the hydrolysis of phosphate groups^68^, hence accelerating the degradation rate with an increase in the concentration of the enzyme. Hydrogels based on polyphosphoesters and PEG have furthermore been shown to be hydrolytically degradable at rates enhanced significantly by the presence of alkaline phosphatase and observed to promote the gene expression of bone‐specific markers.^69^ Meanwhile, polyphosphoesters bearing enzyme‐cleavable acetoxymethyl substituents have been prepared and the substituents cleaved in contact with esterase for 24 h, which although enabling enzymatic switching the polymer properties, does not lead to degradation of the main‐chain.^70^

While there are no reports of polyphosphazene main‐chain itself undergoing enzymatic cleavage, polyphosphazenes with degradation rates accelerated by enzymes have been investigated by our group^71^. Tetrapeptides were added as pendant groups along the polyphosphazene main‐chain. Enzymolysis of the peptides was demonstrated to expose the polyphosphazene backbone leading to accelerated hydrolysis rates of the main‐chain (Figureà12). The polymers were also investigated for triggered delivery of immune response modifiers. However, while drug release was observed, the selectivity towards enzymatic cleavage is not as pronounced as anticipated. This is presumably due to steric shielding of the peptide unit from the enzymes due to folding of the amphiphilic macromolecules, hence may be improved with water soluble peptide sequences.

**Figure 12:**
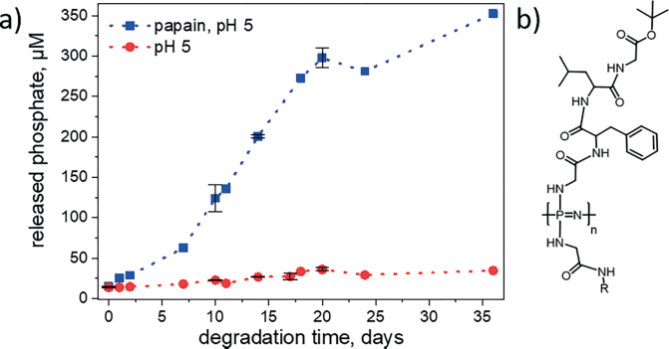
Figure 12. (a) Selective degradation of a peptide functionalized polyphosphazene in the presence of the enzyme papain as observed by a photometric phosphate assay and (b) chemical structure of peptide‐functionalized polyphosphazenes. Adapted from ref.^71^

## 7. Thermoresponsive Polymers

Polymers that undergo sudden conformational transitions in response to small changes in temperature can be termed as thermosensitive or thermoresponsive. One of the most studied such materials are polymers which possess a lower critical solution temperature (LCST) transition in aqueous environments, that is the polymer chains collapse at the LCST leading to precipitation and/or gelation.^72^ When the LCST is around or below body temperature, such polymers become of interest as injectable materials for biomedical applications, including drug delivery and tissue engineering. While commonly based on amphiphilic organic copolymers, whereby the LCST is tuned by the ratio of hydrophobic to hydrophilic moieties, the concept has been successfully translated to a range of phosphorus‐based polymers.

A number of thermoresponsive polyphosphoesters have been prepared, the phosphorus functionalization allowing tuning of the LCST values.^70^, ^74^ For example, controlled ROP polymerization techniques have been exploited to prepare block copolymers^75^. The composition of the polyphosphoesters, via variation of the pendant R group (isopropoxy/ethoxy) on the phosphorus was used to fine tune the LCST. The facile fine‐tuning in combination with biocompatibility and biodegradability represent significant advantages over more well‐known organic‐based thermoresponsive polymers. An alternative, elegant route to fine‐tune the LCST temperatures has been presented by Wurm and co‐workers in which they utilize reversible Diels‐Alder chemistry to add dienophiles to a furan functionalized polyphosphoester main‐chain (Figureà13). The post‐polymerization functionalization allows the amphiphilicity and hence LCST to be readily tuned from a single precursor polymer.^73^ An alternative, facile post‐polymerization method for polyphosphoesters was presented by Wooley and co‐workers[14a] who presented an alkyne functionalized polyphosphoester facilitating functionalization via classical click chemistry methods such as azide–alkyne Huisgen cycloaddition and thiol‐yne addition. The latter has also been used to prepare thermoresponsive polymers, for example poly(d‐glucose carbonate) block copolymers with polyphosphoesters which undergo self‐assembly to form spherical core–shell nanostructures in an LCST driven response^76^ (Figureà14).

**Figure 13:**
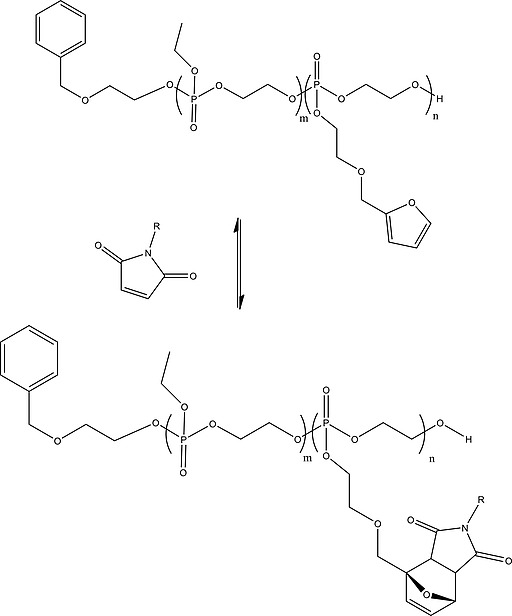
Figure 13. Diels‐Alder functionalization of furan‐functionalized polyphosphoesters as reported in ref.^73^

**Figure 14:**
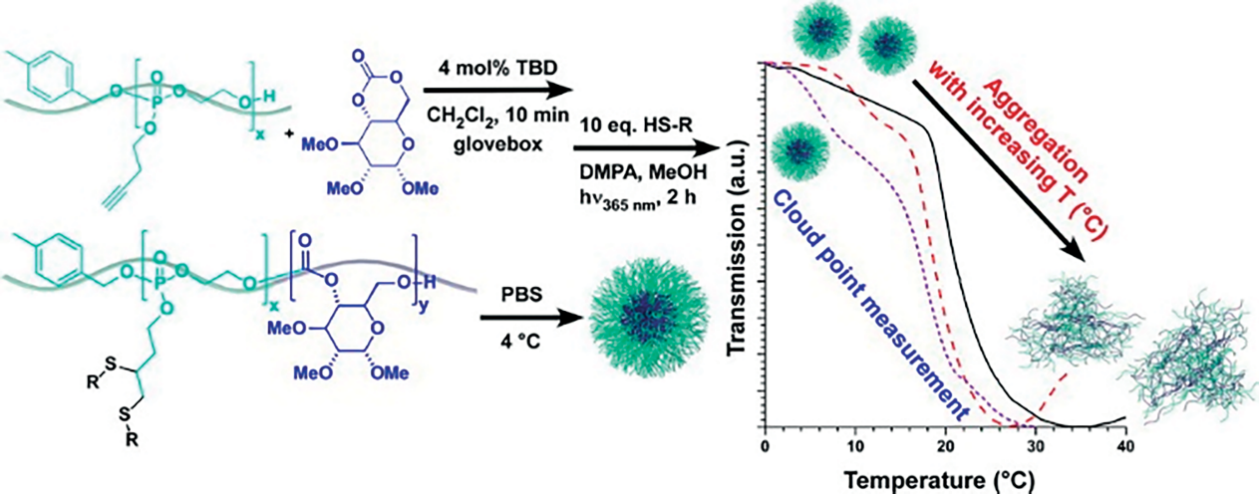
Figure 14. Post‐polymerization functionalization of polyphosphoesters via thiol‐yne addition allows tuning of the amphiphilicity and hence temperature of an LCST driven self‐assembly of the block copolymers. Reproduced with permission from ref.^76^ Copyright 2013 American Chemical Society.

Polyphosphazenes demonstrating thermoresponsive behavior due to an LCST have also been widely reported, the main route being via backbone substitution with amphiphilic oligomers. For example our group showed a simple route to prepare polymers with LCST values in the range of 18 °C to 90 °C^77^ via the post‐polymerization functionalization with Jeffamines (amine‐capped ethylene oxide/ propylene oxide oligomers). The LCST could be tuned depending on the ratio of ethylene oxide to propylene oxide moieties (Figureà15). A similar approach has also been reported with isopropylacrylamide oligomers^78^, effectively translating the well‐known LCST response of PNIPAm to a phosphazene‐based polymer. Furthermore, the co‐substitution of a polyphosphazene backbone with a ratio of different hydrophobic and hydrophilic substituents has been shown to be an efficient method to fine tune the LCST, as well as other important properties of the materials, such as mechanical strength and biodegradability. The property tuning is commonly achieved through a combination of hydrophobic amino acid esters with oligomeric alkoxy poly(ethylene glycol)s (PEG). These materials have been developed into injectable hydrogels by S.‐C. Song and co‐workers and successfully applied in a range of therapeutic targets.^79^

**Figure 15:**
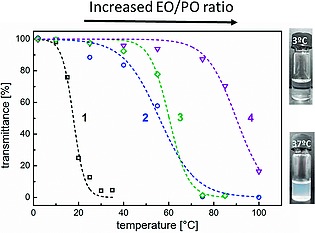
Figure 15. Optical transmittance measurements (500 nm) for aqueous solutions of Jeffamine substituted bottle‐brush polyphosphazenes, demonstrating the tunable LCST behavior. Adapted from ref.^77^

## 8. Conclusions

A broad family of polymers are described, which can respond to several stimuli, either as a direct consequence of phosphorus in the main‐chain or through its use as an easily functionalized connection point for responsive moieties. Although not the only field of use, a major development has been in the biomedical direction due to properties of biodegradability and biocompatibility, a desirable basis for materials intended for this field. In particular, the ability to combine controlled molecular weights with multivalency and post‐polymerization functionalization is important for the stimuli‐responsive materials discussed herein, since it is the chemical functionality and the molecular weight of the polymers that determine the responsive behavior of polymers. The stimuli‐responsive materials detailed herein demonstrate a number of possible gains for phosphorus‐based polymers over classical organic polymers and represent a burgeoning field with still much untapped potential.

## References

[1] M. Wei , Y. Gao , X. Li and M. J. Serpe , Polym. Chem., 2017, 8, 127–143.

[2] a) F. D. Jochum and P. Theato , Chem. Soc. Rev., 2013, 42, 7468–7483; 22868906 10.1039/c2cs35191a

[3] S. Binauld and M. H. Stenzel , Chem. Commun., 2013, 49, 2082–2102. 10.1039/c2cc36589h 23320254

[4] R. Hoogenboom , in: Smart Polymers and their Applications (Eds.: Aguilar M. R. and Román J. San), Woodhead Publishing, 2014, pp. 15–44,

[5] a) C.‐C. Song , F.‐S. Du and Z.‐C. Li , J. Mater. Chem. B, 2014, 2, 3413–3426; 10.1039/c3tb21725f 32261460

[6] J. Thévenot , H. Oliveira , O. Sandre and S. Lecommandoux , Chem. Soc. Rev., 2013, 42, 7099–7116. 23636413 10.1039/c3cs60058k

[7] J. Hu and S. Liu , Macromolecules, 2010, 43, 8315–8330.

[8] L. D. Zarzar and J. Aizenberg , Acc. Chem. Res., 2014, 47, 530–539. 24283993 10.1021/ar4001923

[9] A. Kornberg , N. N. and and D. Ault‐Riché , Annu. Rev. Biochem., 1999, 68, 89–125. 10872445 10.1146/annurev.biochem.68.1.89

[10] a) H. R. Allcock and N. L. Morozowich , Polym. Chem., 2012, 3, 578–590;

[11] a) I. Teasdale and O. Brüggemann , in: Polyphosphazenes for medical applications, Smithers RAPRA, Shrewsbury, UK, 2014;

[12] A.‐M. Caminade , A. Ouali , R. Laurent , C.‐O. Turrin and J.‐P. Majoral , Chem. Soc. Rev., 2015, 44, 3890–3899. 25297494 10.1039/c4cs00261j

[13] a) S. Rothemund and I. Teasdale , Chem. Soc. Rev., 2016, 45, 5200–5215; 27314867 10.1039/c6cs00340k PMC5048340

[14] a) S. Zhang , A. Li , J. Zou , L. Y. Lin and K. L. Wooley , ACS Macro Lett., 2012, 1, 328–333; 22866244 10.1021/mz200226m PMC3410554

[15] S. Zhang , H. Wang , Y. Shen , F. Zhang , K. Seetho , J. Zou , J.‐S. A. Taylor , A. P. Dove and K. L. Wooley , Macromolecules, 2013, 46, 5141–5149. 23997276 10.1021/ma400675m PMC3755629

[16] a) N. Liu , J. Vignolle , J.‐M. Vincent , F. Robert , Y. Landais , H. Cramail and D. Taton , Macromolecules, 2014, 47, 1532–1542;

[17] a) L. Li , Y. Xu , I. Milligan , L. Fu , E. A. Franckowiak and W. Du , Angew. Chem. Int. Ed., 2013, 52, 13699–13702; 10.1002/anie.201306391 24288204

[18] a) A. K. Andrianov and A. Marin , Biomacromolecules, 2006, 7, 1581–1586; 16677042 10.1021/bm050959k

[19] A. K. Andrianov , A. Marin and P. Peterson , Macromolecules, 2005, 38, 7972–7976.

[20] A. Linhardt , M. König , A. Iturmendi , H. Henke , O. Brueggemann , I and Teasdale, Ind. Eng. Chem. Res. 2018, 57, 10, 3602–3609 29568158 10.1021/acs.iecr.7b05301 PMC5857928

[21] S. Rothemund , T. B. Aigner , A. Iturmendi , M. Rigau , B. Husár , F. Hildner , E. Oberbauer , M. Prambauer , G. Olawale , R. Forstner , R. Liska , K. R. Schröder , O. Brüggemann and I. Teasdale , Macromol. Biosci., 2015, 15, 351–363. 25355036 10.1002/mabi.201400390

[22] D. P. DeCollibus , A. Marin and A. K. Andrianov , Biomacromolecules, 2010, 11, 2033–2038. 20690712 10.1021/bm100395u

[23] E. M. Ruiz , C. A. Ramírez , M. A. Aponte and G. V. Barbosa‐Cánovas , Biomaterials, 1993, 14, 491–496. 8329520 10.1016/0142-9612(93)90235-t

[24] J. Baran and S. Penczek , Macromolecules, 1995, 28, 5167–5176.

[25] M. Steinmann , M. Wagner and F. R. Wurm , Chem. Eur. J., 2016, 22, 17329–17338. 27781304 10.1002/chem.201603990

[26] H. Wang , L. Su , R. Li , S. Zhang , J. Fan , F. Zhang , T. P. Nguyen and K. L. Wooley , ACS Macro Lett., 2017, 6, 219–223. 10.1021/acsmacrolett.6b00966 35650917

[27] H. Wang , M. Dong , S. Khan , L. Su , R. Li , Y. Song , Y.‐N. Lin , N. Kang , C. H. Komatsu , M. Elsabahy and K. L. Wooley , ACS Macro Lett., 2018, 7, 783–788. 10.1021/acsmacrolett.8b00377 35650768

[28] S. M. Ibim , A. A. Ambrosio , D. Larrier , H. R. Allcock and C. T. Laurencin , J. Controlled Release, 1996, 40, 31–39.

[29] I. Teasdale , S. Wilfert , I. Nischang and O. Brüggemann , Polym. Chem., 2011, 2, 828–834.

[30] R. Haag and F. Kratz , Angew. Chem. Int. Ed., 2006, 45, 1198–1215; 10.1002/anie.200502113 16444775

[31] E. Markovsky , H. Baabur‐Cohen , A. Eldar‐Boock , L. Omer , G. Tiram , S. Ferber , P. Ofek , D. Polyak , A. Scomparin and R. Satchi‐Fainaro , J. Controlled Release, 2012, 161, 446–460. 10.1016/j.jconrel.2011.12.021 22286005

[32] I. Teasdale and O. Brueggemann , Polymers, 2013, 5, 161–187. 24729871 10.3390/polym5010161 PMC3982046

[33] Z. Zhao , J. Wang , H.‐Q. Mao and K. W. Leong , Adv. Drug Delivery Rev., 2003, 55, 483–499. 10.1016/s0169-409x(03)00040-1 12706047

[34] J. Hu , J. He , D. Cao , M. Zhang and P. Ni , Polym. Chem., 2015, 6, 3205–3216.

[35] C. M. Hackl , B. Schoenhacker‐Alte , M. H. Klose , H. Henke , M. S. Legina , M. A. Jakupec , W. Berger , B. K. Keppler , O. Bruggemann , I. Teasdale , P. Heffeter and W. Kandioller , Dalton Trans., 2017, 46, 12114–12124. 28862707 10.1039/c7dt01767g

[36] Y. Huang , D. Wang , X. Zhu , D. Yan and R. Chen , Polym. Chem., 2015, 6, 2794–2812.

[37] J. Liu , W. Huang , Y. Pang , X. Zhu , Y. Zhou and D. Yan , Biomacromolecules, 2010, 11, 1564–1570. 20364861 10.1021/bm100188h

[38] V. Mitova , S. Slavcheva , P. Shestakova , D. Momekova , N. Stoyanov , G. Momekov , K. Troev and N. Koseva , Eur. J. Med. Chem., 2014, 72, 127–136. 24361525 10.1016/j.ejmech.2013.11.014

[39] R. Duncan and S. C. Richardson , Mol. Pharm., 2012, 9, 2380–2402. 22844998 10.1021/mp300293n

[40] R. Duncan , Curr. Opin. Biotechnol., 2011, 22, 492–501. 21676609 10.1016/j.copbio.2011.05.507

[41] E. Vacchelli , A. Eggermont , W. H. Fridman , J. Galon , L. Zitvogel , G. Kroemer and L. Galluzzi , Oncoimmunology, 2013, 2, e24850. 24073369 10.4161/onci.24850 PMC3782010

[42] S. Aichhorn , A. Linhardt , A. Halfmann , M. Nadlinger , S. Kirchberger , M. Stadler , B. Dillinger , M. Distel , A. Dohnal , I. Teasdale and W. Schöfberger , Chem. Eur. J., 2017, 23, 17721–17726. 28758266 10.1002/chem.201702942 PMC5763314

[43] H. Henke , K. Kryeziu , J. Banfić , S. Theiner , W. Körner , O. Brüggemann , W. Berger , B. K. Keppler , P. Heffeter and I. Teasdale , Macromol. Biosci., 2016, 16, 1239–1249. 27169668 10.1002/mabi.201600035 PMC4976076

[44] S. Suárez‐Suárez , G. A. Carriedo and A. Presa Soto , Chem. Eur. J., 2016, 22, 4483–4491. 26880712 10.1002/chem.201504733

[45] G. A. Carriedo , R. de la Campa and A. P. Soto , Eur. J. Inorg. Chem., 2018, 2018, 2484–2499.

[46] C. Zheng , X. Yao and L. Qiu , Macromol. Biosci., 2011, 11, 338–343. 21104880 10.1002/mabi.201000333

[47] A. P. Martinez , B. Qamar , T. R. Fuerst , S. Muro and A. K. Andrianov , Biomacromolecules, 2017, 18, 2000–2011. 28525259 10.1021/acs.biomac.7b00537 PMC7206414

[48] F. Meng , W. E. Hennink and Z. Zhong , Biomaterials, 2009, 30, 2180–2198. 19200596 10.1016/j.biomaterials.2009.01.026

[49] X. Du , Y. Sun , M. Zhang , J. He and P. Ni , ACS Appl. Mater. Interfaces, 2017, 9, 13939–13949. 28378998 10.1021/acsami.7b02281

[50] C. Chen , P. Zheng , Z. Cao , Y. Ma , J. Li , H. Qian , W. Tao and X. Yang , BioMater. Sci., 2016, 4, 412–417. 26626655 10.1039/c5bm00440c

[51] J. Liu , Y. Pang , J. Chen , P. Huang , W. Huang , X. Zhu and D. Yan , Biomaterials, 2012, 33, 7765–7774. 22818989 10.1016/j.biomaterials.2012.07.003

[52] E. Lallana and N. Tirelli , Macromol. Chem. Phys., 2013, 214, 143–158.

[53] K. E. Broaders , S. Grandhe and J. M. Fréchet , J. Am. Chem. Soc., 2011, 133, 756–758. 21171594 10.1021/ja110468v

[54] F.‐Y. Qiu , C.‐C. Song , M. Zhang , F.‐S. Du and Z.‐C. Li , ACS Macro Lett., 2015, 4, 1220–1224. 10.1021/acsmacrolett.5b00533 35614840

[55] C. de Gracia Lux , S. Joshi‐Barr , T. Nguyen , E. Mahmoud , E. Schopf , N. Fomina and A. Almutairi , J. Am. Chem. Soc., 2012, 134, 15758–15764. 22946840 10.1021/ja303372u PMC3478073

[56] B. W. Rawe , C. P. Chun and D. P. Gates , Chem. Sci., 2014, 5, 4928–4938.

[57] G. I. Peterson , M. B. Larsen and A. J. Boydston , Macromolecules, 2012, 45, 7317–7328.

[58] A. Iturmendi , S. Theis , D. Maderegger , U. Monkowius and I. Teasdale , Macromol. Rapid Commun., 2018, 39, 1800377. 10.1002/marc.201800377 30048024

[59] P. Xiao , J. Zhang , J. Zhao and M. H. Stenzel , Prog. Polym. Sci., 2017, 74, 1–33.

[60] S. Wilfert , A. Iturmendi , W. Schoefberger , K. Kryeziu , P. Heffeter , W. Berger , O. Brüggemann and I. Teasdale , J. Polym. Sci., Part A, 2014, 52, 287–294. 10.1002/pola.27002 PMC3980369 24729657

[61] Z. Li and H. R. Allcock , ACS Appl. Mater. Interfaces, 2015, 7, 13518–13523. 26018938 10.1021/acsami.5b02805

[62] H. R. Allcock , in: Chemistry and Applications of Polyphosphazenes, Wiley, Hoboken, USA, 2003.

[63] T. Potta , C. Chun and S.‐C. Song , Macromol. Rapid Commun., 2010, 31, 2133–2139. 21567641 10.1002/marc.201000350

[64] I. Teasdale , M. Waser , S. Wilfert , H. Falk and O. Brüggemann , Monatsh. Chem., 2012, 143, 355–360.

[65] D. Feinweber , T. Verwanger , O. Brueggemann , I. Teasdale and B. Krammer , Photochem. Photobiol. Sci., 2014, 13, 1607–1620. 25257955 10.1039/c4pp00251b

[66] F. Li , C. Chen , X. Yang , X. He , Z. Zhao , J. Li , Y. Yu , X. Yang and J. Wang , ACS Appl. Mater. Interfaces, 2018, 10, 21198–21205. 29897728 10.1021/acsami.8b06758

[67] Y.‐C. Wang , L.‐Y. Tang , T.‐M. Sun , C.‐H. Li , M.‐H. Xiong and J. Wang , Biomacromolecules, 2008, 9, 388–395. 18081252 10.1021/bm700732g

[68] C. Wachiralarpphaithoon , Y. Iwasaki and K. Akiyoshi , Biomaterials, 2007, 28, 984–993. 17107708 10.1016/j.biomaterials.2006.10.024

[69] D.‐A. Wang , C. G. Williams , F. Yang , N. Cher , H. Lee and J. H. Elisseeff , Tissue Eng., 2005, 11, 201–213. 15738675 10.1089/ten.2005.11.201

[70] I. Yasuhiko , K. Takashi and Y. Shin‐ichi , Chem. Lett., 2009, 38, 1054–1055.

[71] A. Linhardt , M. König , W. Schöfberger , O. Brüggemann , A. Andrianov and I. Teasdale , Polymers, 2016, 8, 161. 10.3390/polym8040161 PMC6432119 30979252

[72] Q. Zhang , C. Weber , U. S. Schubert and R. Hoogenboom , Mater. Horiz., 2017, 4, 109–116.

[73] G. Becker , T. A. Marquetant , M. Wagner and F. R. Wurm , Macromolecules, 2017, 50, 7852–7862.

[74] a) Y. Iwasaki and E. Yamaguchi , Macromolecules, 2010, 43, 2664–2666;

[75] Y.‐C. Wang , L.‐Y. Tang , Y. Li and J. Wang , Biomacromolecules, 2009, 10, 66–73. 19133835 10.1021/bm800808q

[76] T. P. Gustafson , A. T. Lonnecker , G. S. Heo , S. Zhang , A. P. Dove and K. L. Wooley , Biomacromolecules, 2013, 14, 3346–3353. 23957247 10.1021/bm4010832

[77] S. Wilfert , A. Iturmendi , H. Henke , O. Brüggemann and I. Teasdale , Macromol. Symp., 2014, 337, 116–123. 24926189 10.1002/masy.201450314 PMC4050288

[78] J. X. Zhang , L. Y. Qiu , K. J. Zhu and Y. Jin , Macromol. Rapid Commun., 2004, 25, 1563–1567.

[79] a) B.‐B. Seo , H.‐I. Chang , H. Choi , J.‐T. Koh , K.‐D. Yun , J.‐Y. Lee and S.‐C. Song , J. Biomed. Mater. Res. Part B, 2018, 106, 751–759; 10.1002/jbm.b.33885 28334520

